# Report of the Proceedings of the National Medical Convention

**Published:** 1870-05

**Authors:** 


### Report of the Proceedings of the National Medical Convention.

In the very full account given in the present number of the *Journal*, of the proceedings of the late meeting of the American Medical Association, we have quoted from the "*Washington Morning Chronicle*," the "*Evening Star*," and the "*Boston Medical Journal.*"

